# Transcriptomic data sets for *Novosphingobium aromaticivorans* grown with the β-5-linked aromatic dimer dehydrodiconiferyl alcohol and the related G-aromatic monomers vanillin and ferulic acid

**DOI:** 10.1128/mra.00845-24

**Published:** 2024-10-04

**Authors:** Fletcher Metz, Kevin S. Myers, Fachuang Lu, Daniel R. Noguera, Timothy J. Donohue

**Affiliations:** 1 DOE Great Lakes Bioenergy Research Center, University of Wisconsin, Madison, Wisconsin, USA; 2 Wisconsin Energy Institute, University of Wisconsin, Madison, Wisconsin, USA; 3 Laboratory of Genetics, University of Wisconsin, Madison, Wisconsin, USA; 4 Department of Civil and Environmental Engineering, University of Wisconsin, Madison, Wisconsin, USA; 5 Department of Bacteriology, University of Wisconsin, Madison, Wisconsin, USA; SUNY College of Environmental Science and Forestry, Syracuse, New York, USA

**Keywords:** genomics, genetics, ligninolysis, aromatic compounds

## Abstract

The transcriptomes of a 2-pyrone-4,6-dicarboxylic acid-producing strain of *Novosphingobium aromaticivorans* DSM12444 were determined when grown in minimal medium containing glucose alone or glucose plus vanillin, ferulic acid, or the β-5-linked aromatic dimer dehydrodiconiferyl alcohol as carbon sources. Here, we present the RNA-sequencing data we obtained.

## ANNOUNCEMENT

The Alphaproteobacterium *Novosphingobium aromaticivorans* DSM12444 is a promising chassis bacterium for lignin valorization due to its ability to funnel various aromatic compounds (1–3) into a central aromatic metabolic pathway that can be engineered for the production of commodity chemicals, such as 2-pyrone-4,6-dicarboxylic acid (PDC) (3, 4) and *cis-cis-*muconic acid (5). To valorize lignin, the interunit bonds that join aromatics must be cleaved. We recently elucidated the pathway by which this bacterium catabolizes aromatics joined by the second most abundant interunit linkage in lignin, the β-5 linkage (6, 7), using the model dimer dehydrodiconiferyl alcohol (DC-A) (8). We identified candidate DC-A catabolism genes by conducting an RNA-Seq experiment, in which we grew a PDC-producing strain of *N. aromaticivorans* (PDC12444) (3) in standard mineral base (SMB) minimal medium (9) supplemented with glucose alone or glucose plus vanillin, ferulic acid, or DC-A. Vanillin and ferulic acid were chosen as control aromatic carbon sources because they are aromatic monomers that we hypothesized to be downstream intermediates of DC-A catabolism that are funneled into central aromatic metabolism through known pathways (3, 10). Transcriptomic data for these conditions were collected as previously described (8) and briefly detailed below.

Four overnight *N. aromaticivorans* PDC12444 cultures were diluted 1:1 with SMB minimal medium supplemented with 1 g/L glucose and grown for 1 hour at 30°C. The cultures were then each diluted 1:100 into separate cultures of SMB minimal medium supplemented with 1 g/L glucose alone or 1 g/L glucose plus 0.5 mM DC-A, 0.5 mM vanillin, or 0.5 mM ferulic acid. These cultures were incubated at 30°C for 5–6 hours until they reached mid-exponential growth phase (determined based on pilot experiments). The cells were pelleted by centrifugation and stored at −80°C. They were later thawed, lysed, and RNA was extracted using hot acid phenol:chloroform extraction, as previously described (11). The samples were treated with RNase-free DNase, and RNA was purified using the RNeasy Kit (Qiagen, Germantown, MD).

RNA-Seq library preparation and sequencing were performed by the Joint Genome Institute (JGI) using default parameters. RNA in the samples was depleted using the QIAseq FastSelect Kit (Qiagen, Germantown, MD), and libraries were constructed using the TruSeq Stranded mRNA Kit (Illumina, San Diego, CA) following standard JGI protocols. The libraries were sequenced on an Illumina NovaSeq to produce 2 × 150 reads. Reads were trimmed using Trimmomatic version 0.3 with the default settings except for a HEADCROP of 5, LEADING of 3, TRAILING of 3, SLIDINGWINDOW of 3:30, and MINLEN of 36 (12). After trimming, the reads were aligned to the *N. aromaticivorans* DSM12444 genome sequence (GenBank accession GCF_000013325.1) using bwa-mem (version 0.7.17-h5bf99c6_8) with default settings (13). Alignment files were further processed with Picard-tools (version 2.26.10) (https://broadinstitute.github.io/picard/) (*CleanSAM* and *AddOrReplaceReadGroups* commands) and samtools (version 1.2) (*sort* and *index* commands) (14). Paired aligned reads were assigned to genes using HTSeq version 0.6.0 (15). Raw sequencing reads were normalized using the fragments per kilobase per million mapped reads method.

RNA-Seq data sets have proven useful for studying metabolism in *N. aromaticivorans* (8, 16). Our transcriptomic data sets (Table 1) will enable investigators to identify the transcriptomic responses of *N. aromaticivorans* to DC-A and related aromatic monomers.

**TABLE 1:** Summary of RNA-Seq sample data

Sample	Carbon source(s)	Replicate number	GEO accession number	SRA accession number
Glucose Rep 1	Glucose	1	GSM8114835	SRX23772439
Glucose Rep 2	Glucose	2	GSM8114836	SRX23772447
Glucose Rep 3	Glucose	3	GSM8114837	SRX23772448
Glucose Rep 4	Glucose	4	GSM8114838	SRX23772449
DC-A Rep 1	DC-A and glucose	1	GSM8114839	SRX23772450
DC-A Rep 2	DC-A and glucose	2	GSM8114840	SRX23772451
DC-A Rep 3	DC-A and glucose	3	GSM8114841	SRX23772452
DC-A Rep 4	DC-A and glucose	4	GSM8114842	SRX23772453
Vanillin Rep 1	Vanillin and glucose	1	GSM8114843	SRX23772454
Vanillin Rep 2	Vanillin and glucose	2	GSM8114844	SRX23772440
Vanillin Rep 3	Vanillin and glucose	3	GSM8114845	SRX23772441
Vanillin Rep 4	Vanillin and glucose	4	GSM8114846	SRX23772442
Ferulic Acid Rep 1	Ferulic acid and glucose	1	GSM8114847	SRX23772443
Ferulic Acid Rep 2	Ferulic acid and glucose	2	GSM8114848	SRX23772444
Ferulic Acid Rep 3	Ferulic acid and glucose	3	GSM8114849	SRX23772445
Ferulic Acid Rep 4	Ferulic acid and glucose	4	GSM8114850	SRX23772446

## Data Availability

Raw RNA-Seq reads are available from NCBI SRA (PRJNA1081366), and gene expression data are available in NCBI GEO (GSE259365).

## References

[1] Fredrickson JK, Brockman FJ, Workman DJ, Li SW, Stevens TO. 1991. Isolation and characterization of a subsurface bacterium capable of growth on toluene, naphthalene, and other aromatic compounds. Appl Environ Microbiol 57:796–803. doi:10.1128/aem.57.3.796-803.1991 16348445 PMC182797

[2] Fredrickson JK, Balkwill DL, Drake GR, Romine MF, Ringelberg DB, White DC. 1995. Aromatic-degrading Sphingomonas isolates from the deep subsurface. Appl Environ Microbiol 61:1917–1922. doi:10.1128/aem.61.5.1917-1922.1995 7544095 PMC167454

[3] Perez JM, Kontur WS, Alherech M, Coplien J, Karlen SD, Stahl SS, Donohue TJ, Noguera DR. 2019. Funneling aromatic products of chemically depolymerized lignin into 2-pyrone-4-6-dicarboxylic acid with. Green Chem 21:1340–1350. doi:10.1039/C8GC03504K

[4] Perez JM, Sener C, Misra S, Umana GE, Coplien J, Haak D, Li YD, Maravelias CT, Karlen SD, Ralph J, Donohue TJ, Noguera DR. 2022. Integrating lignin depolymerization with microbial funneling processes using agronomically relevant feedstocks. Green Chem 24:2795–2811. doi:10.1039/D1GC03592D

[5] Vilbert AC, Kontur WS, Gille D, Noguera DR, Donohue TJ. 2024. Engineering Novosphingobium aromaticivorans to produce cis,cis-muconic acid from biomass aromatics. Appl Environ Microbiol 90:e0166023. doi: 10.1128/aem.01660-23 38117061 10.1128/aem.01660-23 PMC10807440

[6] Guadix-Montero S, Sankar M. 2018. Review on catalytic cleavage of C–C inter-unit linkages in lignin model compounds: towards lignin depolymerisation. Top Catal 61:183–198. doi:10.1007/s11244-018-0909-2

[7] Chen Z, Wan CX. 2017. Biological valorization strategies for converting lignin into fuels and chemicals. Renew Sustain Energy Rev 73:610–621. doi:10.1016/j.rser.2017.01.166

[8] Metz F, Olsen AM, Lu F, Myers KS, Allemann MN, Michener JK, Noguera DR, Donohue TJ. 2024. Catabolism of β-5 linked aromatics by Novosphingobium aromaticivorans. MBio 15:e0171824. doi:10.1128/mbio.01718-24 39012147 PMC11323797

[9] Kontur WS, Bingman CA, Olmsted CN, Wassarman DR, Ulbrich A, Gall DL, Smith RW, Yusko LM, Fox BG, Noguera DR, Coon JJ, Donohue TJ. 2018. Novosphingobium aromaticivorans uses a Nu-class glutathione S-transferase as a glutathione lyase in breaking the β-aryl ether bond of lignin. J Biol Chem 293:4955–4968. doi:10.1074/jbc.RA117.001268 29449375 PMC5892560

[10] Cecil JH, Garcia DC, Giannone RJ, Michener JK. 2018. Rapid, parallel identification of catabolism pathways of lignin-derived aromatic compounds in Novosphingobium aromaticivorans. Appl Environ Microbiol 84:e01185-18. doi:10.1128/AEM.01185-18 30217841 PMC6210112

[11] Lakey BD, Myers KS, Alberge F, Mettert EL, Kiley PJ, Noguera DR, Donohue TJ. 2022. The essential Rhodobacter sphaeroides CenKR two-component system regulates cell division and envelope biosynthesis. PLoS Genet 18:e1010270. doi:10.1371/journal.pgen.1010270 35767559 PMC9275681

[12] Bolger AM, Lohse M, Usadel B. 2014. Trimmomatic: a flexible trimmer for Illumina sequence data. Bioinformatics 30:2114–2120. doi:10.1093/bioinformatics/btu170 24695404 PMC4103590

[13] Li H, Durbin R. 2009. Fast and accurate short read alignment with Burrows-Wheeler transform. Bioinformatics 25:1754–1760. doi:10.1093/bioinformatics/btp324 19451168 PMC2705234

[14] Li H, Handsaker B, Wysoker A, Fennell T, Ruan J, Homer N, Marth G, Abecasis G, Durbin R, Genome Project Data Processing S. 2009. The sequence alignment/Map format and SAMtools. Bioinformatics 25:2078–2079. doi:10.1093/bioinformatics/btp352 19505943 PMC2723002

[15] Anders S, Pyl PT, Huber W. 2015. HTSeq--a python framework to work with high-throughput sequencing data. Bioinformatics 31:166–169. doi:10.1093/bioinformatics/btu638 25260700 PMC4287950

[16] Linz AM, Ma Y, Perez JM, Myers KS, Kontur WS, Noguera DR, Donohue TJ. 2021. Aromatic dimer dehydrogenases from Novosphingobium aromaticivorans reduce monoaromatic diketones. Appl Environ Microbiol 87:e0174221. doi:10.1128/AEM.01742-21 34613756 PMC8612281

